# The influence mechanism of planned behavior and self-consciousness on learners’ willingness to use language learning Apps: Validation based on structural equation modeling

**DOI:** 10.1371/journal.pone.0319300

**Published:** 2025-03-28

**Authors:** Yanyan Ren, Xiaoyong Tan

**Affiliations:** 1 School of Foreign Languages and Literatures, Chongqing Normal University, Chongqing, People’s Republic of China; 2 College English Education Center, Chongqing Normal University, Chongqing, People’s Republic of China; Chulalongkorn University, Thailand

## Abstract

The continuous development of digital technology facilitates the informatization of foreign language learning, making it possible to create a deeply integrated mobile learning environment which is conducive to enhancing learning efficiency. To investigate learners’ willingness to use mobile language leaning applications (Apps), this research is carried out under an extended theoretical framework of Technology Acceptance Model (TAM) and Theory of Planned Behavior (TAM) by incorporating self-consciousness. On the basis of the survey data of 728 college students, structural equation modeling is constructed and empirically tested with AMOS 23.0. Results show that the structural equation modeling based on the extended TAM-TPB framework is suitable for analyzing the influencing mechanism of learners’ willingness to use language learning Apps. College students’ willingness to use language learning Apps is directly affected by behavioral attitudes, subjective norms, and perceived behavior control. Perceived ease of use has a significant direct impact on perceived usefulness. However, perceived ease of use and perceived usefulness have different mediating effects on learners’ willingness to use language learning Apps through behavioral attitudes, subjective norms, and perceived behavior control. Based on the findings, this paper suggests that when developing language learning Apps, emphasis should be laid on the positive feedback of learners’ self-consciousness and user enjoyment, as well as the construction of a curriculum system centered on core competencies so as to enhance learners’ willingness to use language learning Apps, thereby promoting language learning efficiency.

## 1. Introduction

With the development of mobile networks and the rapid growth of mobile terminal users, various types of mobile applications (Apps) have become the main tools for communication and information acquisition. According to the “Statistical Report on the Development of China’s Internet Network” (https://www.cnnic.cn/n4/2023/0828/c88-10829.html), as of June 2023, the number of internet users in China had reached 1.079 billion, an increase of 11.09 million over December 2022. Among them, 99.6% had access to the internet though mobile phones. The number of active Apps monitored in the market was 2.6 million. In China, Apps, as one of the main products of the mobile internet, are developing rapidly, not only in huge quantities, but also in wide varieties, among which game Apps, health Apps, learning Apps, and daily communication Apps have become the most popular ones.

Supported by application technology and equipment improvement, an increasing amount of mobile application scenarios are being developed and a wide variety of Apps have emerged in the field of academic research. Database publishers have developed academic Apps with various functions, including Apps that provide database access, e-books/journals, and Apps for professional and transactional functions. In addition, technology companies and research institutions have also developed Apps that either provide academic information or serve as academic communities [1]. Meanwhile, due to the impact of public emergencies, online learning has become one of the main modes for teaching and doing research. It has been found that the large-scale deployment and application of 5G communication networks, as well as their integration with artificial intelligence, virtual reality, and cloud computing, will enable online education platforms based on 5G technology to better meet the diverse and personalized learning needs of learners [2]. Whether used as a “substitute” for offline learning when it cannot be conducted due to external limitations or as a means to improve learners’ learning ability under new learning environment, the importance of online learning cannot be underestimated, or rather, it will play an even more pivotal role in future learning [3].

Mobile learning is becoming a major mode of online learning, with various types of educational Apps emerging in large numbers. According to Blake et al., if used appropriately, technology can play an important role in enhancing second language learners’ exposure to other cultures and languages [4]. However, learners’ willingness to use mobile learning Apps differs greatly [5], and the analysis of the mechanism influencing their willingness to use language learning Apps has become a key research direction. Many researches have been carried out on factors influencing users’ willingness to use different types of Apps. Wang et al. constructed a model to examine users’ willingness to adopt hotel booking mobile applications based on the VAM model theory [6]. With a survey of health App users, it has been concluded that personal health awareness directly affects users’ willingness to use health Apps [7], which shows the importance of self-awareness in users’ App adaptation willingness. Structural equation modeling is frequently applied to study users’ willingness to use mobile applications. For example, it is used to study the influencing mechanism of users’ willingness to use live streaming Apps [8], the degree of farmers’ acceptance of agricultural mobile applications [9]. In studies concerning teaching and learning Apps, conclusions have been drawn that perceived enjoyment, flexibility, and self-regulation positively affect user satisfaction with mobile learning Apps, which in turn can increase learners’ willingness for continuous use [10]. Individual creativity, perceived usefulness, and perceived enjoyment have a significant positive impact on learners’ willingness to use mobile learning Apps, and are the basis and prerequisite for improving mobile learning performance [11]. For college students, willingness to use learning Apps is affected by factors such as their personal experience, perceived ease of use and perceived usefulness of the Apps, as well as the university’s online learning support and the teachers’ online teaching proficiency [12].

Review of existing literature shows that most of the previous studies explore people’s acceptance of new technologies and their willingness to use them under the framework of Technology Acceptance Model (TAM), while studies concerning the impact of individual behavioral differences on their willingness to use Apps, especially language learning Apps, are rare. The Theory of Planned Behavior (TPB) holds that human behavior is the result of deliberate planning, i.e., human behavior is influenced by behavioral attitudes, subjective norms, perceived behavioral control, and behavioral intentions, which can help to explain how people change their behavioral patterns. Therefore, taking an integrated theoretical framework of TAM and TPB (TAM-TPB) as the theoretical model and introducing individual self-consciousness as an important external dependent variable affecting learners’ willingness to use language learning Apps, this study explores the influencing mechanism of leaners’ willingness to use language learning Apps with validation based on structural equation modeling.

## 2. Theoretical basis and research hypotheses

### 2.1 Theoretical basis

The various actions taken by users to obtain and use the goods or services are defined as user behavior, which represents a certain purpose of use. “Awareness - familiarization - trail- use - loyalty” is the typical process of user behavior. The earliest research on user behavior can be traced back to the Theory of Reasoned Action proposed by M. Fishbein and Ajzen, which is used to analyze how attitudes consciously affect individual behavior, with a focus on the process of attitude formation based on cognitive information. On this basis, scholars have continuously extended and revised the theory, based on which TAM, TPB, and UTAUT (Unified Theory of Acceptance and Use of Technology), etc. have been proposed.

Among them, TAM is often used to study users’ acceptance of information systems, which is mainly affected by perceived usefulness (PU) and perceived ease of use (PEOU) of the technology. TPB holds that the individual’s behavioral intention and behavior are the result of deliberate planning, which are jointly influenced by attitudes, subjective norms, and perceived behavioral control [13], and it is often used to explain and predict behavioral intention. Since inception, TAM and TPB have demonstrated high explanatory and predictive power in explaining the theoretical relationship between users’ attitude and behavior towards accepting and using information technology.

Language learning Apps are an IT-based learning tool, and users’ willingness to use them is influenced by both technology acceptance and behavioral attitudes. When explaining user willingness, the integrated framework of TAM and TPB, i.e., TAM-TPB, is of higher explanatory power than TAM or TPB alone [14]. On the one hand, TAM can effectively measure the influencing factors of learners’ acceptance of new technologies. On the other hand, the use of language learning Apps is not only a behavior controlled by individual will and behavioral intention, but also constrained by individual ability, opportunity and resources, making it suitable to be studied within the framework of TPB. Therefore, based on the integrated theoretical framework of TAM-TPB, this study constructs a conceptual model to verify the influencing effects of factors, namely, perceived usefulness, perceived ease of use, behavioral attitudes, subjective norms, and perceived behavior control on learners’ willingness to use language learning Apps.

Self-consciousness is individuals’ cognition, experience and desire to their physical and mental states, which has the characteristics of purposefulness and dynamicity and plays an important role in regulating, monitoring and correcting the formation and development of personality. Therefore, incorporating self-consciousness in the model will expand the study of behavioral intention and more effectively explain learners’ willingness to use language learning Apps.

### 2.2 Research hypotheses

(1) Influence of perceived usefulness and perceived ease of use on learners’ willingness to use language learning Apps

With regard to the use of language learning Apps, theorists who support the application of technology emphasize the use of the most advanced technology to enhance operational performance. However, language learning is more about application scenarios, and the development and use of Apps should focus on enhancing the effectiveness of knowledge learning. TAM focuses on exploring the role of the two main determinants, i.e., perceived usefulness and perceived ease of use, in behavioral intention. Perceived usefulness reflects individuals’ perception that using language learning Apps makes the learning process more interactive, which has a positive impact on their learning performance. Perceived ease of use reflects how easy individuals think it is to use technology [15] and thereby influencing their perception of its usefulness. Undoubtedly, individuals usually adopt more positive attitudes towards using a new technology if they believe it is easy to use and can bring benefits [16]. In the use of language learning Apps, perceived ease of use mainly comes from learners’ mastery of the technological tools and their evaluation of the complexity of the technological platform. Perceived usefulness may directly affect learners’ willingness to use language learning Apps. Learner’s perceived ease of use can affect their perceived usefulness because when they believe the language learning Apps are convenient for information retrieval and for conducting learning activities; in other words, when they have a relatively high level of perceived ease of use, they are more likely to perceive the Apps to be useful and thus more willing to use them. Based on the above analysis, the following hypotheses are proposed:

H1: Perceived ease of use has a significant positive impact on perceived usefulness of language learning Apps.
H2: Perceived ease of use has a significant positive impact on learners’ willingness to use language learning Apps.

(2) Influence of behavioral attitudes, subjective norms and perceived behavioral control on learners’ willingness to use language learning Apps

According to TPB, an individual’s behavior intention and behavior are the result of deliberate planning and are influenced by behavioral attitudes, subjective norms, and perceived behavior control. Behavioral attitudes reflect learners’ preferences and assessment of the value for the use of language learning Apps. With positive behavioral attitudes, learners are more likely to have behavioral intention. Subjective norms reflect the influence of other individuals, organizations, or social groups’ expectations on learners in the learning process [17]. When learners perceive more social pressure for using an App, they tend to show stronger willingness to use it, demonstrating the influence of external factors on learners’ willingness to use technology. Perceived behavior control reflects learners’ judgment on the application of technology based on past experiences and anticipated obstacles. Learners with stronger perceived behavior control ability are more capable of controlling external factors, leading to stronger willingness to use language learning Apps. Research has also shown that perceived behavioral control has a significant positive effect on behavioral intention [18]. Based on the above analysis, the following hypotheses are proposed:

H3: Behavioral attitudes have a significant positive impact on learners’ willingness to use language learning Apps.
H4: Subjective norms have a significant positive impact on learners’ willingness to use language learning Apps.
H5: Perceived behavior control has a significant positive impact on learners’ willingness to use language learning Apps.

(3) Influence of self-consciousness on learners’ perceived ease of use and perceived usefulness of language learning Apps

Self-consciousness refers to the degree to which attitudinal tendencies are integrated into daily activities. Individuals with high self-consciousness usually approach life and learning with more positive attitudes, and they are more likely to allocate time rationally and mobilize their knowledge reserves to adapt to the application of new technology. The primal function of language learning Apps is to help learners develop language skills, thereby expanding or constructing their knowledge system. Learners are influenced by many cognitive factors when using Apps, and their self-consciousness and willingness to use the technological tools are intertwined. Therefore, it is especially necessary to take self-consciousness as an external variable and explore its impact on learners’ perceived ease of use and perceived usefulness of language learning Apps. Learning is not a “disembodied” mental training, but an “embodied” cognitive construction [19]. Self-consciousness is one of the essential, formative, and motivational conditions for learner’s formation of self-perception [20], and it is also the main influencing factor of learning behavior. Learners with higher levels of self-consciousness are more likely to acknowledge that technological advances have a significant impact on learning. Based on the above analysis, the following hypotheses are proposed:

H6: Self-consciousness has a significant positive impact learners’ perceived ease of use of language learning Apps.
H7: Self-consciousness has a significant positive learners’ perceived usefulness of language learning Apps.

(4) Transmission mechanism of influencing factors

Based on TAM, perceived usefulness and perceived ease of use are important factors affecting learners’ willingness to use language learning Apps. However, since perceived ease of use and perceived usefulness are subjective feelings of different users, they need to be reinforced continuously by certain behaviors in order to generate behavior intention. When learners find that using language learning Apps is conducive to improving learning efficiency, their perceived usefulness of using technology will increase, which in turn will promote positive attitudes towards using the Apps. According to TPB, perceived behavioral control reflects an individual’s perception of whether certain factors facilitate or hinder a particular behavior, depending on their perception of how easy or difficult to perform the behavior [21]. When learners believe a technological tool is easy to use, they will perceive themselves to have a high degree of control over the behavior and their expectations of the consequences of the behavior will be more aligned, thereby enhancing their willingness to use the technology. Subjective norms refer to users’ cognition of the surrounding usage environment, including the exemplary guidance from other users, which are less susceptible to the influence of individuals’ subjective usability evaluation and are more likely to be improved as a result of personal usage gains. In other words, subjective norms are more likely to be influenced by users’ perceived usefulness of a technological tool, which can enhance their subjective evaluation, improve their behavioral attitudes, and thus increase their willingness to use the tool. Based on the above analysis, the following hypotheses are proposed:

H8: Subjective norms have a significant positive impact on behavioral attitudes.
H9: Perceived ease of use has a significant positive impact on behavioral attitudes.
H10: Perceived ease of use has a significant positive impact on perceived behavior control.
H11: Perceived usefulness has a significant positive impact on behavioral attitudes.
H12: Perceived usefulness has a significant positive impact on subjective norms.
H13: Perceived usefulness has a significant positive impact on perceived behavior control.

**Fig 1:**
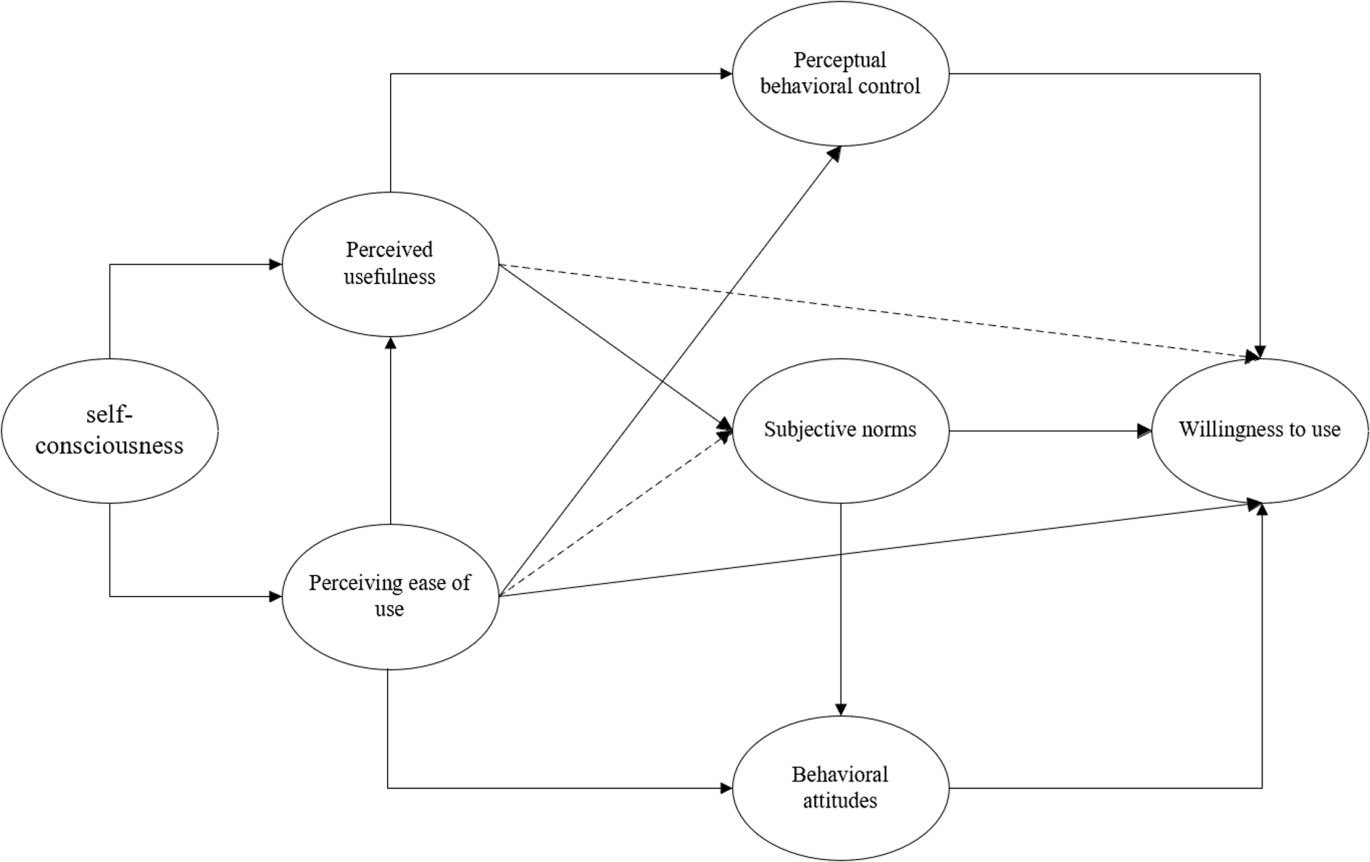
Conceptual model of learners’ willingness to use language learning Apps.

Based on the above hypotheses, this study constructs a conceptual model of the influencing factors of learners’ willingness to use language learning Apps within the theoretical framework of TAM-TPB, as shown in Fig 1:

## 3. Data and variables

### 3.1 Data sources

In order to investigate the effect of self-consciousness and factors of TPB, i.e., behavioral attitudes, subjective norms and perceived behavioral control, on learners’ willingness to use language learning Apps, a questionnaire is designed and distributed through the online survey platform “Wenjuanxing” from March 8, 2022 to May 6, 2022. The survey targets college students, covering non-foreign language majors specializing in engineering, science, and humanities and social sciences, and all of them are adults, over 18 years old. Respondents are informed of the purpose of the survey and they grant verbal consent to participation before filling out the questionnaire, which has been reported to and approved by the ethics committee. A total of 750 questionnaires are collected, and after removing the incomplete ones, 728 questionnaires are obtained as valid for analysis. Among the valid questionnaire respondents, 40.93% are male students and 59.07% are female students. Since foreign language courses are mainly offered to first- and second-year undergraduate students, students of this group account for 89.97%. Besides, 36.54% of the valid questionnaire respondents are engineering majors, 40.66% are science majors, and 22.80% major in humanities and social sciences. The sample composition meets the requirements of representativeness and diversity.

### 3.2 Variable selection and description

In this study, learners’ perceived ease of use of language learning Apps is examined from the aspects of convenience for registration, convenience for information retrieval and access as well as convenience for coursework submission, while learners’ perceived usefulness is examined in terms of whether language learning Apps can ensure interaction, improve learning efficiency, save learning costs and provide learners with diversified and personalized learning contents. Learners’ behavioral attitudes towards language learning Apps are examined based on their belief of the convenience, comfort and enjoyment when using language learning Apps. Subjective norms of learners’ perception of language learning Apps are investigated in terms of other learners’ usage, recognition and recommendations. The perceived behavior control of language learning Apps is examined from the perspectives of learners’ controllability and confidence in the Apps. Learners’ willingness to use language learning Apps is investigated through their willingness to try, to share their experience and to make recommendations.

Self-consciousness is learners’ self-assessment of their physical and mental activities, which is measured with the “Self-Consciousness Scale” developed by psychologists Fenigstein, Sctrier and Buss. According to the characteristics of learners’ use of technology, 8 indicators, namely, behavioral assessment, self-reflection, individual performance, inner feelings, sensitivity to emotional changes, self-interpretation, motivation, and psychological evaluation, are selected for measurement. Variables involved in the study are all measured with a 5-point Likert scale, where 1 represents “Strongly Disagree” and 5 represents “Strongly Agree”. The relationship between variables is investigated numerically for the purpose of empirical analysis. Descriptive statistics of the data are shown in Table 1.

**Table1:** Variable selection and descriptive statistics.

Latent variables	Observed variables	Variable code	Mean	Standard deviation
self-consciousness (SC)	I care about the way I do things.	SC1	3.67	0.573
	I often reflect on myself.	SC2	3.53	0.647
	I care about how I behave.	SC3	3.69	0.516
	I am concerned about how I feel.	SC4	3.75	0.487
	I am sensitive to the changes in my mood.	SC5	3.64	0.592
	I often try to express myself.	SC6	3.33	0.799
	I often examine my own motivations.	SC7	3.46	0.691
	I am aware of my mental status when solving problems.	SC8	3.49	0.679
Perceived ease of use (PE)	The registration procedure for the language learning Apps is simple.	PE1	3.65	1.087
	It is easy to search and obtain information using language learning Apps.	PE2	3.75	1.011
	It is convenient to submit assignments using language learning Apps.	PE3	3.84	0.985
Perceived usefulness (PU)	Interaction can be achieved through using language learning Apps.	PU1	3.26	1.105
	Learning efficiency can be promoted by using language learning Apps.	PU2	3.30	1.089
	Learning costs can be saved by using language learning Apps.	PU3	3.69	1.032
	I can choose what I want to learn by using language learning Apps.	PU4	3.74	1.005
Behavioral attitudes (BA)	Learning with language learning Apps is convenient.	BA1	3.68	0.994
	Learning with language learning Apps is comfortable.	BA2	3.47	1.068
	Learning with language learning Apps is enjoyable.	BA3	3.35	1.062
Subjective norms (SN)	Most of my classmates use language learning Apps.	SN1	3.66	1.039
	Most of my classmates believe it is necessary to use language learning Apps.	SN2	3.37	1.010
	Teachers in our university recommend using language learning Apps.	SN3	3.66	0.901
Perceived behavioral control (BC)	I am very handy in using language learning Apps.	BC1	3.29	0.985
	I have the ability to make better use of language learning Apps.	BC2	3.30	1.003
	I am confident that I can make better use of language learning Apps.	BC3	3.24	1.051
Willingness to use (UW)	Under current conditions, I am willing to continue to use/try to use language learning Apps.	UW1	3.53	1.032
	I would like to share my experience of using language learning Apps.	UW2	3.08	1.121
	I am willing to recommend other students to use language learning Apps.	UW3	3.32	1.100

### 3.3 Reliability and validity of the data

The reliability of the survey data is tested with Cronbach’s alpha coefficient, and the data validity is tested with KMO (Kaiser-Meyer-Olkin) and Bartlett spherical test value (Bartlett). SPSS 27.0 is used to process the data. Results show that the KMO value is 0.941 and the Cronbach’s alpha is 0.946, indicating that the data are of high validity and reliability [22]. The result of Barrett spherical test shows P < 0.05, which manifests that the Self-consciousness Scale is of good internal consistency and suitable for factor analysis. The construct reliability (CR) value of all the indicators is above 0.70, indicating that the variables are of high construct reliability.

## 4. Empirical analysis

### 4.1 Modeling and results

**Fig 2:**
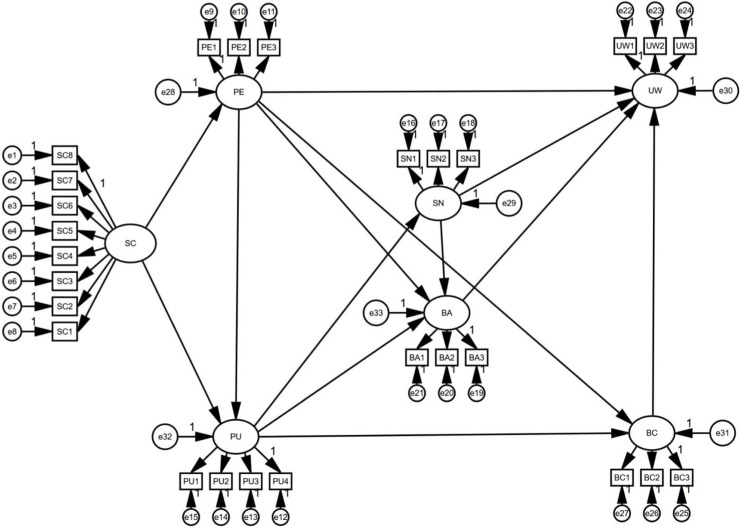
Structural equation modeling of learners’ willingness to use language learning Apps.

In this paper, a structural equation modeling as shown in Fig 2 is established, and AMOS 23.0 is used for model fitting. Results show that all the fitting indexes meet the requirements, among which the Root Mean Square Error of Approximation (RMSEA = 0.079 < 0.08), Tucker-Lewis index (TLI = 0.969 > 0.9), Comparative Fit index (CFI = 0.984 > 0.9), GFI, AGFI, IFI, CFL, and PGFI meet the recommended value standards, indicating that the model fits well. Standardized regression coefficients after model fitting are shown in Table 2 and the mediating effect relationship is shown in Table 3.

**Table 2:** Structural equation modeling analysis.

Paths	Estimate	S.E.	C.R.	P	Standardized Estimate
PE < ---SC	0.675	0.026	26.064	^***^	0.363
PU < ---SC	0.216	0.018	11.714	^***^	0.117
PU < ---PE	0.707	0.015	46.455	^***^	0.712
SN < ---PU	0.786	0.016	50.322	^***^	0.766
BC < ---PU	1.075	0.019	55.403	^***^	0.808
BA < ---PU	0.661	0.022	29.59	^***^	0.476
BC < ---PE	0.177	0.015	11.955	^***^	0.134
BA < ---SN	0.112	0.016	7.157	^***^	0.083
BA < ---PE	0.572	0.017	32.885	^***^	0.415
UW < ---SN	0.208	0.013	16.46	^***^	0.162
UW < ---BA	0.243	0.013	18.236	^***^	0.255
UW < ---PE	0.007	0.016	0.440	0.660	0.005
UW < ---BC	0.583	0.012	48.334	^***^	0.587
SC8 < ---SC	1				0.563
SC7 < ---SC	1.033	0.025	41.809	^***^	0.572
SC6 < ---SC	0.934	0.027	34.683	^***^	0.447
SC5 < ---SC	0.954	0.022	44.011	^***^	0.616
SC4 < ---SC	0.938	0.019	49.203	^***^	0.736
SC3 < ---SC	1.055	0.021	50.858	^***^	0.782
SC2 < ---SC	1.114	0.024	45.956	^***^	0.658
SC1 < ---SC	1.073	0.022	48.440	^***^	0.717
PE1 < ---PE	1				0.654
PE2 < ---PE	1.303	0.019	67.607	^***^	0.916
PE3 < ---PE	1.02	0.017	58.995	^***^	0.736
PU4 < ---PU	1				0.702
PU3 < ---PU	1.109	0.016	67.241	^***^	0.758
PU2 < ---PU	1.374	0.018	78.343	^***^	0.89
PU1 < ---PU	1.274	0.018	71.983	^***^	0.814
SN1 < ---SN	1				0.697
SN2 < ---SN	1.248	0.018	68.096	^***^	0.895
SN3 < ---SN	0.786	0.015	53.408	^***^	0.632
BA3 < ---BA	1				0.924
BA2 < ---BA	1.021	0.007	156.306	^***^	0.938
BA1 < ---BA	0.886	0.007	128.088	^***^	0.873
UW1 < ---UW	1				0.908
UW2 < ---UW	0.918	0.01	92.563	^***^	0.766
UW3 < ---UW	1.032	0.008	122.080	^***^	0.879
BC3 < ---BC	1				0.894
BC2 < ---BC	1.008	0.007	145.283	^***^	0.945
BC1 < ---BC	0.948	0.007	129.956	^***^	0.903

* P < 0.05;

** P < 0.01;

*** P < 0.001.

**Table 3:** Two tailed significance of bias-corrected percentile method.

	Bias-corrected Percentile Method 95% CI				Percentile Method 95% CI			
Parameter	Estimate	Lower	Upper	P	Estimate	Lower	Upper	P
PE < --- SC	0.675	0.622	0.717	0.013	0.675	0.624	0.718	0.01
PU < --- SC	0.216	0.185	0.248	0.009	0.216	0.184	0.248	0.01
PU < --- PE	0.707	0.675	0.757	0.006	0.707	0.672	0.751	0.01
SN < --- PU	0.786	0.748	0.827	0.009	0.786	0.747	0.826	0.01
BC < --- PU	1.075	1.015	1.122	0.018	1.075	1.019	1.127	0.01
BA < --- PU	0.661	0.618	0.73	0.004	0.661	0.61	0.715	0.01
BC < --- PE	0.177	0.136	0.221	0.009	0.177	0.135	0.219	0.01
BA < --- SN	0.112	0.081	0.139	0.021	0.112	0.085	0.144	0.01
BA < --- PE	0.572	0.529	0.619	0.012	0.572	0.529	0.621	0.01
UW < --- SN	0.208	0.172	0.239	0.02	0.208	0.181	0.241	0.01
UW < --- BA	0.243	0.212	0.277	0.007	0.243	0.211	0.275	0.01
UW < --- PE	0.007	-0.03	0.054	0.749	0.007	-0.029	0.054	0.749
UW < ---BC	0.583	0.547	0.614	0.009	0.583	0.546	0.613	0.01

As can be seen in Table 2, the statistical results show that the influence of each observed variable on the latent variables is significant. As for the influence paths, the p-value of UW < ---PE is 0.060, indicating that perceived ease of use has no significant impact on leaners’ willingness to use language learning Apps, while the p-values of all other influence paths are below 0.001, indicating that the impacts are significant. Therefore, all the other 12 hypotheses except H2 are confirmed.

From Table 3, it can be found that 0 is excluded in the lower and upper intervals of the bias-corrected 95% CI except for that of UW < --- PE, and the same is true in the lower and upper intervals of the percentile 95% CI. The p-values for the bias-corrected 95% CI and percentile 95% CI are less than 0.05, indicating the existence of mediating effects in the influence paths. Detailed analysis will be made in 4.2.

### 4.2 Response path analysis of learners’ willingness to use language learning apps

(1) Self-consciousness

Self-consciousness is not only an individual’s knowledge about himself/herself, but also recognition of his/her own identity, including abstraction, self-representation, representation of other individuals, and action. More importantly, it is also the learning process of self-representation through direct learning (based on personal experience) and observational learning (based on observation of other individuals) [23]. Results of the structural equation modeling show that self-consciousness has a significant promoting effect on learners’ perceived ease of use and perceived usefulness of language learning Apps, with the impact coefficients of 0.363 and 0.117, respectively. That is, the stronger self-consciousness, the stronger learners’ perceived ease of use and perceived usefulness of language learning Apps, which verifies H6 and H7. This may be due to the fact the self-consciousness encompasses the thinking process from motivation to cognition and execution, which can help learners understand their own learning styles and learning habits, enabling them to choose the most suitable learning methods and new technology application methods to improve learning efficiency. Consequently, their evaluation of the language learning Apps will be improved. At the same time, learning with Apps demonstrates a multimodal feedback framework assisted by big data visualization, which provides teachers with a digital workflow to effectively use multimodal feedback [24]. Then, college students are more likely to obtain immediate and positive feedback, thereby increasing their perceived ease of use and perceived usefulness.

(2) Perceived ease of use

For perceived ease of use, the convenience of using language learning Apps for information retrieval and access is higher than that for submitting assignments and registration and the path coefficients of PE2 < ---PE, PE3 < ---PE, PE1 < ---PE decrease sequentially (0.916 > 0.736 > 0.654), which clearly shows that learners’ perceived ease of use of language learning Apps is most affected by convenience for information retrieval and access. Meanwhile, it can be seen from the model results the paths of PU < ---PE, BA < ---PE, BC < ---PE are significant, in other words, in the response mechanism, perceived ease of use has a significant positive facilitating effect on perceived usefulness, behavioral attitudes and perceived behavior control, which validates H2, H9 and H10. This also confirms that using social media for collaborative learning has a direct positive impact on perceived usefulness and perceived ease of use [25], and college students’ perception of the ease of using mobile learning tools will affect their perception of the usefulness of mobile learning [26]. However, perceived ease of use has no significant effect on college students’ willingness to use language learning Apps, that is, perceived ease of use is not related to their willingness to use technological tools [27]. The reason is probably that college students are well-educated and usually have stronger awareness of mobile information security [28]. Their learning ability is relatively high and using technological tools is not a big challenge to them. Therefore, perceived ease of use is only an indicator of their evaluation of language learning Apps, rather than a facilitating factor for their willingness to use them, although it is widely recognized that if the language learning Apps are considered difficult to use, they are more likely to be abandoned.

(3) Perceived usefulness

Whether the use of language learning Apps can improve learning efficiency has the greatest impact on perceived usefulness, with a coefficient of 0.890, indicating that learners are more concerned about the improvement of learning efficiency when using language learning Apps. This also confirms that college students have the awareness of using information technology products for learning [29]. Meanwhile, the model results also show that the paths of SN < ---PU, BC < ---PU, BA < ---PU are significant, that is, in the response mechanism, perceived usefulness has a significant positive facilitating effect on subjective norms, behavioral attitudes and perceived behavior control, which validates H11, H12 and H13. Further analysis reveals that perceived usefulness has the greatest impact on perceived behavior control, with a coefficient of 0.808, higher than that on subjective norms (0.766) and behavioral attitudes (0.476). This is mainly because perceived usefulness means that learners consider technological tools as useful in promoting problem-solving and optimizing the learning process [30], thus strengthening their perceived behavioral control. The promoting effect of perceived usefulness on behavioral attitudes and subjective norms validates the learning process from cognition, feedback to implementation. In addition, perceived usefulness plays a mediating role between information quality or source reliability and user behavior [31], which also validates the path that self-consciousness ultimately influences learners’ willingness to use language learning Apps through perceived usefulness and perceived ease of use.

(4) Subjective norms

Subjective norms have a significant facilitating effect on learners’ willingness to use language learning Apps, that is, the higher the subjective norms of college students towards technological tools, the higher their willingness to use them. This verifies H4 and also conforms to the finding that subjective norms affect learners’ intention to register in flipped classrooms [32], manifesting that subjective norms have a significant positive effect on learners’ willingness to engage in technology-based autonomous learning. For individuals, subjective norms are the result of environmental shaping, and the formation process is influenced and strengthened by exemplary norms and mandatory norms, which in turn shape individuals’ behavioral attitudes. This is consistent with the model results that subjective norms have a significant positive impact on behavioral attitudes, thus validating H8.

(5) Behavioral attitudes

In this study, convenience, enjoyment and comfort are used to depict the tendency of behavioral attitudes, with the coefficient of enjoyment (0.938) higher than that of convenience (0.924) and comfort (0.873), indicating that college students’ behavioral attitudes towards language learning Apps are most affected by whether they can derive pleasure from using the Apps. This is mainly because positive emotional feedback is the most important factor in shaping behavioral attitudes. Results of the model clearly show when learners have more positive behavioral attitudes, they are more likely to use language learning Apps. This validates H3 and corresponds to the research conclusion that college students’ attitudes significantly affect their willingness to use the teaching resource databases [33], as well as confirms the positive predictive effect of learning attitudes on learning intention concerning using language learning technological tools [34].

(6) Perceived behavior control

Perceived behavioral control is a subjective perception which can have an impact on individual behavior. It refers to individuals’ expectations regarding the relationship between factors like their personality and behavior and upcoming or already occurred events. These expectations are often generalized and “expectation reinforcement” plays a significant role in regulating behavior [35]. Perceived behavioral control is an essential human need and a major driving force for human behavior. It directly affects college students’ willingness to use language learning Apps, as the use of Apps is actually a cognitive process that involves the understanding and processing of external information and the ultimate decision-making. The improvement of cognitive ability can help learners better plan and trigger actions. The model results demonstrates that perceived behavioral control significantly and positively affects learners’ willingness to use language learning Apps, which validates H5 and corresponds to the findings that perceived behavioral control directly influences users’ willingness to use artificial intelligence learning platforms [36].

## 5 Conclusions and recommendations

In the digital era, new language learning modes are constantly emerging, one of which is learning through Apps. Under such circumstances, it is necessary to continuously innovate and improve the teaching mode and adjust the teaching content on the basis of analyzing the behavioral patterns of contemporary college students so as to meet their learning needs and help them enhance learning efficiency. Within the integrated theoretical framework of TAM-TPB, this paper validates the promoting effect of self-consciousness on learners’ perceived ease of use and perceived usefulness of language learning Apps, as well as the positive impact and mediating effect of behavioral attitudes, subjective norms and perceived behavior control on learners’ App application willingness. Based on the study, the following recommendations are proposed.

(1) Highlight the positive feedback of learners’ self-consciousness and strengthen its effectiveness in facilitating pedagogical exploration. In the process of teaching design and the application of technological tools, proactive measures can be taken to improve learners’ awareness of cognition, evaluation and feedback, which is potentially positive for the effective use of language learning Apps. Based on the positive promoting effect of self-consciousness, learning evaluation should shift from traditional horizontal evaluation to vertical evaluation, emphasizing the diachronic comparison of learning efficiency. This can effectively help learners understand themselves, build up confidence, enhance learning enthusiasm, and become active practitioners of learning, thus improving learning performance.
(2) Emphasize user enjoyment in the functional design of language learning Apps. In the process of learning system construction, gamification can be reasonably applied, which helps learners to enhance perceived ease of use and perceived usefulness, thereby promoting their endorsement of the new learning mode and willingness to use technological tools. Besides, attention should be paid to the influence of behavioral patterns on learners’ technology using willingness. When designing language learning Apps, interactive modules that integrate viewing, listening and speaking should be added to create virtual language learning scenarios to ensure sufficient input and output, which are essential crucial for language learning. All these will help increase learners’ engagement in learning, deepen their understanding of the learning content, and promote their learning effectiveness.
(3) Construct a curriculum system centered on core competencies. Reasonable, flexible, targeted, and creative learning modes often have a profound impact on learners. Learning through Apps is conducive to breaking down learners’ abstract understanding of language learning, shaping their behavioral attitudes and subjective norms, and strengthening the demonstrative effect of App usage. As a result, learners will be more willing to use language learning Apps. Meanwhile, structurally embedding curriculum implementation and evaluation and emphasizing the regulatory role of “expectation reinforcement” on behavior will also promote learners’ perceived behavior control and their willingness to use technological tools.
